# Individual Differences in Different Measures of Opioid Self-Administration in Rats Are Accounted for by a Single Latent Variable

**DOI:** 10.3389/fpsyt.2021.712163

**Published:** 2021-09-07

**Authors:** Yayi Swain, Niels G. Waller, Jonathan C. Gewirtz, Andrew C. Harris

**Affiliations:** ^1^Hennepin Healthcare Research Institute, Minneapolis, MN, United States; ^2^Department of Psychology, University of Minnesota, Minneapolis, MN, United States; ^3^Department of Neuroscience, University of Minnesota, Minneapolis, MN, United States; ^4^Department of Medicine, University of Minnesota, Minneapolis, MN, United States

**Keywords:** opioid self-administration, factor analysis, individual differences, behavioral economics, multivariate methods

## Abstract

Individual differences in vulnerability to addiction have been widely studied through factor analysis (FA) in humans, a statistical method that identifies “latent” variables (variables that are not measured directly) that reflect the common variance among a larger number of observed measures. Despite its widespread application in behavioral genetics, FA has not been used in preclinical opioid addiction research. The current study used FA to examine the latent factor structure of four measures of i.v. morphine self-administration (MSA) in rats (i.e., acquisition, demand elasticity, morphine/cue- and stress/cue-induced reinstatement). All four MSA measures are generally assumed in the preclinical literature to reflect “addiction vulnerability,” and individual differences in multiple measures of abuse liability are best accounted for by a single latent factor in some human studies. A one-factor model was therefore fitted to the data. Two different regularized FAs indicated that a one-factor model fit our data well. Acquisition, elasticity of demand and morphine/cue-induced reinstatement loaded significantly onto a single latent factor while stress/cue-induced reinstatement did not. Consistent with findings from some human studies, our results indicated a common drug “addiction” factor underlying several measures of opioid SA. However, stress/cue-induced reinstatement loaded poorly onto this factor, suggesting that unique mechanisms mediate individual differences in this vs. other MSA measures. Further establishing FA approaches in drug SA and in preclinical neuropsychopathology more broadly will provide more reliable, clinically relevant core factors underlying disease vulnerability in animal models for further genetic analyses.

## Introduction

Individual differences in susceptibility to addiction in humans have been studied widely through factor analysis (FA), a statistical method that identifies “latent” variables (variables that are not measured directly) that reflect the common variance among a larger number of observed measures. In contrast to “bottom-up” approaches evaluating a wide range of measures e.g. (1), FA is a theory-driven statistical method that uses well-defined indicators from a common behavioral domain (2). These models provide both insights into the relationship between different facets of addiction- and dependence-related symptomatology (3), and a relatively parsimonious account of disease comorbidity (4). For example, FA approaches have revealed that liability to alcohol abuse is associated both with a general drug abuse vulnerability factor and with several factors that are specific to this form of addiction (e.g., genetic variants in alcohol metabolizing enzymes) (5–7).

Factor analytic approaches have been widely used in the clinical literature to explore the factor structure underlying various addiction measures. Such structures may have both vertical and horizontal dimensions. The vertical dimension essentially represents hierarchical relationships between concrete traits or behaviors and higher-order, more abstract, or general “latent” factors. The horizontal dimension represents the degree of similarity between factors within a single level of the hierarchy (8). Elaboration of such two-dimensional factor structure may yield one or more robust endophenotypes that can be used to identify genomic loci associated with core features of substance use disorders (9, 10).

In animal addiction research, FA approaches could be useful in identifying the underlying associations between, and uniqueness of, different addiction-related behavioral measures, developing more reliable measures of addiction, and uncovering their underlying genomic and neurobiological substrates (11). Such approaches have rarely been employed in this area, however, despite the fact that the drug self-administration (SA) paradigm models a variety of aspects of addiction (e.g., acquisition, relapse, etc.) within individual subjects, thereby lending itself to multivariate statistical analyses. In one previous study, an exploratory FA revealed three addiction vulnerability measures—(a) SA despite punishment, (b) progressive ratio (PR) breakpoint, and (c) drug-seeking during no-drug periods—as loading onto a single latent factor underlying cocaine SA in rats, whereas extinction loaded onto a separate factor (12). However, despite the dramatic impact of opioid addiction on public health (13), no preclinical studies have applied FA to opioid SA.

The primary goal of this study was to use FA to examine the latent factor structure between four measures of i.v. opioid (morphine) SA in rats (i.e., acquisition, demand elasticity, morphine/cue-induced reinstatement, stress/cue-induced reinstatement), using data from a previously published study (14). The four SA measures were selected due to their common use in preclinical studies and to the relevance of each to different aspects of addiction (15–18).

In animal research, there is frequently the implicit assumption that a variety of different SA variables all have relevance to addiction vulnerability. This is consistent with findings in humans showing that individual differences in multiple measures of abuse liability are best accounted for by a single latent factor (19–21). Therefore, in the current study, a one-factor model was fitted to the data, with the single latent factor conceptualized as the “addiction” factor.

It has been proposed that the minimum sample size required for FA ranges between *N* = 50–250 (22–26). Conducting small sample-size FA may result in many issues that are otherwise uncommon in large sample-size analyses, such as Heywood cases denoting negative estimated variances (22, 27). Preclinical addiction studies have typically employed relatively small sample sizes, which pose a challenge to the use of FA. Therefore, the secondary goal of this study was to test the utility of a novel approach to conducting FA on preclinical data that allows for smaller sample sizes to be used. Several proposed (28–30) “regularization methods” can effectively address the challenges of conducting small sample FA by reducing the number of estimated model parameters. In this study, we utilized two robust regularization methods in conjunction with a method to obtain a robust correlation matrix from our data (31) to demonstrate the feasibility of conducting FA in a small preclinical dataset. By applying these iterative statistical procedures to our data, we aimed to understand the core dimensions underlying the morphine SA (MSA) model.

## Materials and Methods

### Overview of Experimental Protocol

**Figure 1:**
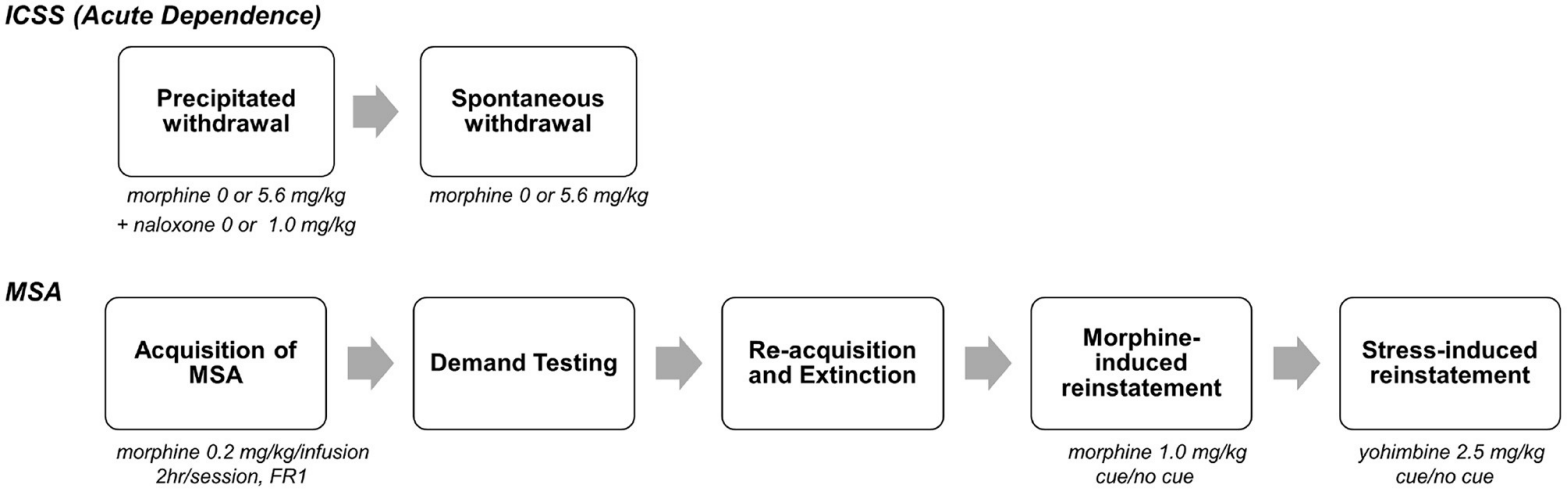
Overview of experimental protocol. On each day, rats were injected with morphine (0 or 5.6, mg., s.c.), followed 1 h 50 min later by naloxone (0 or 1.0 mg/kg), and then tested for ICSS 10 min later. After precipitated withdrawal, rats were injected with morphine (0 or 5.6 mg/kg) and tested for ICSS at multiple time points (2–170 h) after injection. After completion of spontaneous withdrawal testing, all animals were tested using various measures of MSA (e.g., acquisition, demand, reinstatement) in daily 2 h sessions (phase 2). [See text and (14) for further details]. The current FA study used data from MSA (phase 2). Because MSA was not influenced by treatment during ICSS testing (14), all animals that completed the MSA protocol were included in this analysis (*N* = 43). Portion of figure reprinted by permission from Springer Nature (14), copyright 2020.

Data from a recent study (14) were used for the current analyses. The goal of that study was to evaluate whether withdrawal-induced anhedonia as measured using elevated intracranial self-stimulation (ICSS) thresholds predicted individual differences in subsequent MSA. Figure 1 shows an overview of the experimental protocol, which is described in detail in Swain et al. (14). Briefly, male Sprague-Dawley rats that were trained in an ICSS paradigm underwent naloxone (NX)-precipitated and/or spontaneous withdrawal from acute morphine (MOR) injections or received control (saline, SAL) injections. This resulted in four experimental groups: MOR + NX (*n* = 29), MOR + SAL, SAL + NX, SAL + SAL (*n* = 10–11 each). During the subsequent MSA protocol, all rats acquired MSA (0.2 mg/kg/infusion) under a fixed ratio (FR) 1 schedule of reinforcement for at least 10 daily sessions and until MSA was stable. Rats then underwent demand testing in which the FR response requirement was progressively increased every 3–4 sessions as follows: FR 2, 3, 6, 12, 24, and doubled thereafter until infusion rates were reduced by >90% compared to FR 1. Rats then re-acquired MSA under an FR 1 schedule for at least 5 sessions and until infusions/session were stable and subsequently underwent extinction of MSA in the absence of the cue light previously paired with morphine infusions for at least 10 sessions and until active lever pressing was stable. Rats were then tested for drug-induced reinstatement (with morphine injection prior to the SA session) and finally, stress-induced reinstatement (with injection of the pharmacological stressor yohimbine prior to the SA session), both in the presence and absence of the visual cue paired with morphine, and with appropriate within-subject control conditions (1 session per experimental/control condition) [see (14) for more details on animals, apparatus and experimental protocol]. Since a history of MOR and/or NX injections during ICSS testing did not have a significant effect on subsequent MSA, rats from all groups that completed all phases of the study were included in the data analyses (*N* = 43). These experiments conformed to appropriate NIH and institutional ethical / biosafety standards see (14).

### Overview of Factor Model

We tested a one-factor model with one latent variable (the “addiction” factor) and four observed variables from the MSA model: acquisition, elasticity of demand (α), and morphine/cue- and stress/cue-induced reinstatement with visual cue light present. These measures were chosen due to the distinct aspects of addiction-like behavior they are often thought to capture and their common application in drug SA research. Acquisition was defined as the average number of infusions across the first 10 days of MSA. An exponential demand function was fitted to data from the FR escalation protocol to obtain the α statistic, as described in previous studies (14, 16). α refers to the rate of change in consumption with increases in unit price (elasticity of demand), with higher α values indicating lower reinforcement efficacy. Reinstatement was measured as the difference between the number of active and inactive lever presses over each of the 2-h reinstatement test sessions after the challenge (i.e., morphine or yohimbine) drug injection, with cue light present. The use of difference scores to measure reinstatement controls for potential non-specific (e.g., motoric) effects of treatments (14, 32–34). These reinstatement conditions were analyzed because they produced more robust reinstatement than either the challenge drug (morphine or yohimbine) alone or the cue alone (see Results). A higher number of infusions during acquisition, lower elasticity of demand (α), and higher reinstatement scores reflect greater abuse liability for each of these measures.

### Statistical Analyses

All statistical analyses were performed in GraphPad Prism (GraphPad Software, San Diego, California USA) and R ver. 4.0.4 (35). A one-factor model was hypothesized to show good model-fit with each of the SA measures showing high factor loadings, indicating a common “addiction” factor underlying all tested SA measures.

Three distinct methods were used for extracting factor loadings. Given the small sample size of our data set and several outlying values (to be discussed later), we used two distinct factor extraction algorithms that are known to yield robust factor loadings in small sets of non-normal data. The first method involves computing Mahalanobis distances for all data points and then identifying the number of multivariate outliers via a series of chi-squared tests (α = 0.1). Next, we used the minimum covariance determinant [MCD: MASS package (31, 36)] method to produce a robust estimator of multivariate scatter and center to remove the multivariate outliers and generate a robust correlation matrix. This robust correlation matrix was factor analyzed with a regularized least squares estimator [fareg function; (37)]. Robust least squares estimation does not assume data multinormality and aims to minimize residuals between the observed and reproduced correlations under the proposed factor model (38). Model fit was tested via the correlation root mean square residual (CRMR) statistic:


$$\begin{array}{l} CRMR=\sqrt{{\frac{1}{t-p}\underset{i<j}{\sum }\left(\rho_{ij}-\rho_{ij}^{0}\right)}^{2}}, \end{array}$$

with *t* denoting the number of non-redundant population variances and co-variances among the *p* observed variables, ρ_*ij*_ denoting the correlation between variables *i* and *j*, and $\rho_{ij}^{0}$ denoting the model-implied population correlation under the theoretical model (39). CRMR is commonly used in FA and structural equation modeling (SEM) as a model fit statistic, with smaller numbers indicating better model fit. Finally, effect size of overall model misfit was determined by the Γ_1_ statistic, defined as


$$\begin{array}{l} \Gamma_{1}=\frac{p}{{tr\left({\sum \sum_{0}}^{-1}\right)}^{2}}, \end{array}$$

where Σ denotes a population covariance matrix, Σ_0_ denotes the population covariance under the null hypothesis, and *tr* denotes the trace operator (the trace of a square matrix equals the sum of its diagonal elements). Parametric bootstrap standard errors (SE) were computed for the factor loadings using 5,000 bootstrap samples (40).

The second robust method for analyzing the data used regularized FA as described by Jung et al. [(29, 30); implemented in the fareg function; (37)]. Both least squares (LS) and maximum likelihood (MLE) regularized FA were used to estimate robust factor loadings for testing the 1-factor model. Previous work suggests that these methods work well in small samples of non-normally distributed data (27, 29, 30) and thus were well-suited for the current study.

To further demonstrate the advantages of the robust correlation and robust factor analytic methods, a third analysis was implemented using principal axis factoring, a traditional factor extraction method, with the complete data set of 43 rats [using the faMain function in the R fungible library (37)]. This method was not expected to perform well given the small sample size of our data set and the existence of several multivariate outliers. To allow comparison with the other analyses, we also computed the CRMR index for this analysis.

## Results

### MSA

**Figure 2:**
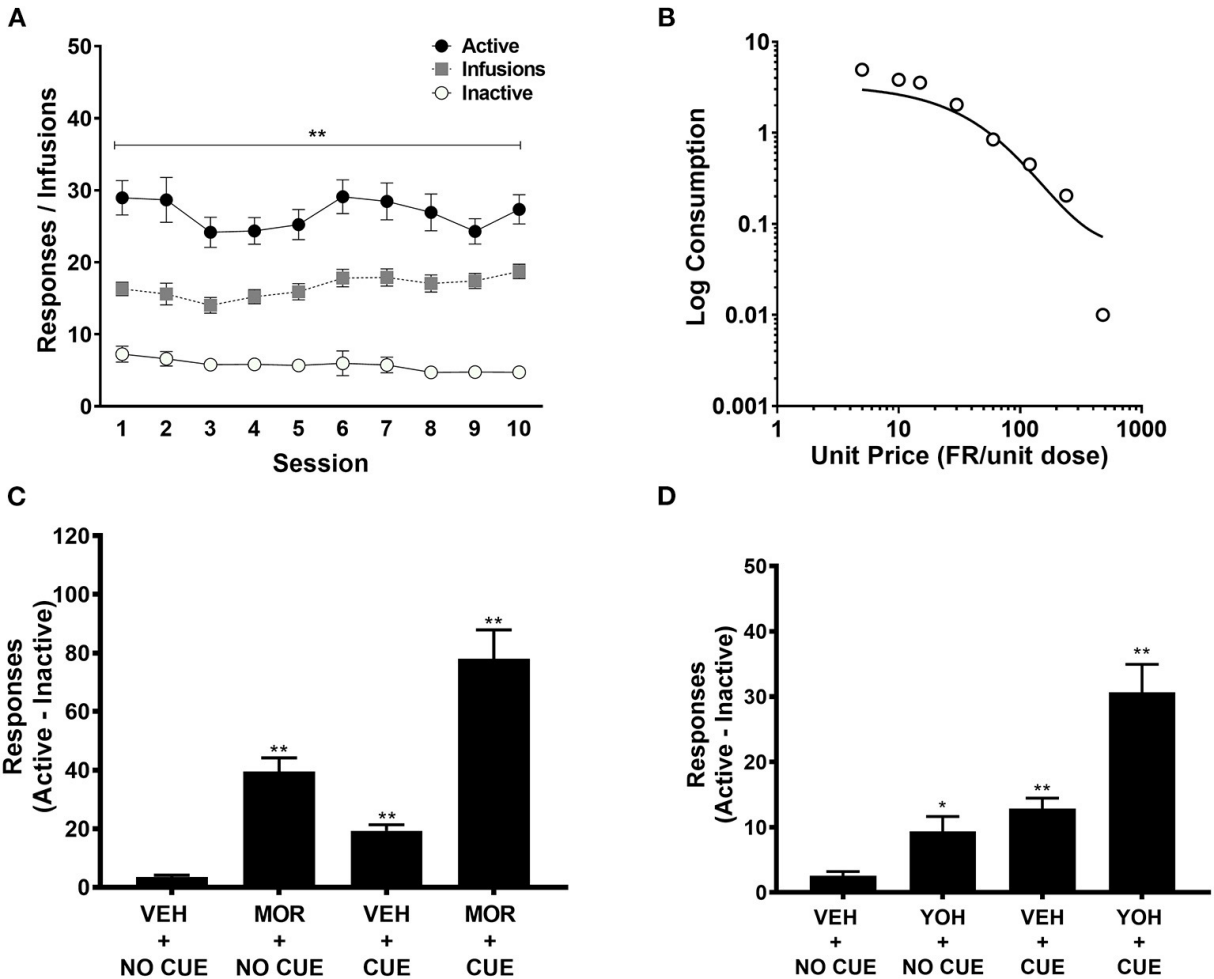
Active and inactive lever pressing during acquisition (*n* = 43), **(A)**; exponential demand curve for morphine intake during demand testing (*n* = 43) **(B)**; difference scores between active and inactive lever pressing during morphine-induced (*n* = 43) **(C)** and yohimbine-induced (*n* = 43) **(D)** reinstatement. These data are derived from Swain et al. (14), but are here pooled across groups irrespective of treatment prior to MSA. MOR, Morphine; YOH, Yohimbine; VEH, Vehicle. Data points represent mean ± SEM. *Significant difference compared to inactive lever pressing or VEH+NO CUE responding, *p* < 0.05; **p < 0.01.

Detailed behavioral results from the MSA protocol are reported in Swain et al. (14). Briefly, rats reliably acquired MSA, exhibiting a clear preference for the active over inactive response lever (Figure 2A). Increases in FR requirement resulted in a progressive reduction in morphine consumption that was well-described by an exponential demand function (*R*^2^ = 0.84) (Figure 2B). After MSA reacquisition and extinction in the absence of the morphine-associated cue light, rats reliably reinstated active lever responding following a priming dose of morphine in the absence of the cue light (MOR + NO CUE; Figure 2C), response-contingent presentation of the cue light (VEH + CUE), or combined exposure to morphine and the cue light (MOR + CUE). Similar findings were observed when reinstatement was induced by the pharmacological stressor yohimbine (Figure 2D).

### FA

All variables were standardized to keep their scales consistent. The factor loadings from each analysis are shown in Table 1.

**Table 1:** Estimates for factor loadings from 3 analyses, with bootstrapped standard errors for the factor loadings from the two robust methods in parenthesis.

	**Factor loadings**		
**SA measures**	**Robust LS**	**Robust MLE**	**Principal axis**
Acquisition	0.58 (0.14)	0.59 (0.08)	0.48
Demand	−0.63 (0.14)	−0.64 (0.13)	−1.03
Morphine/cue-induced reinstatement	0.62 (0.14)	0.63 (0.14)	0.32
Stress/cue-induced reinstatement	0.27 (0.22)	0.28 (0.23)	0.27

*Robust LS, regularized FA using least squares estimates with MCD robust correlation matrix excluding 5 multivariate outliers; Robust MLE, regularized FA using maximum likelihood estimates with robust correlation matrix excluding 5 multivariate outliers; Principal Axis, traditional principal axis factor extraction*.

For the two regularized FA analyses, 5 multivariate outliers (α = 0.1) were identified from the chi-squared test using Mahalanobis distance. Subsequently, these 5 multivariate outliers were excluded from the robust correlation matrix computation using MCD (*N* = 38) (31). Using the robust correlation matrix with LS estimation, the first regularized FA revealed that acquisition, elasticity of demand and morphine/cue-induced reinstatement showed high factor loadings (all |loadings| ≥ 0.58) on a single common factor, whereas stress/cue-induced reinstatement showed low factor loading on this dimension (loading = 0.27) (Table 1). Bootstrap SEs for factor loadings of acquisition, elasticity of demand and morphine/cue-induced reinstatement were also lower (SE = 0.14 for all three) compared to SE of the factor loading for stress/cue-induced reinstatement (SE = 0.22). The second regularized FA using MLE factoring (on the same robust correlation matrix) produced similar results. Acquisition, elasticity of demand and morphine/cue-induced reinstatement showed high factor loadings on a single dimension (all |loadings| ≥ 0.59), and stress/cue-induced reinstatement again showed a low factor loading (loading = 0.28) (Table 1). Similar SEs were also observed, where acquisition, elasticity of demand and morphine/cue-induced reinstatement showed lower SEs (all SEs ≤ 0.14) compared to stress/cue-induced reinstatement (SE = 0.23). Overall, based on the CRMR and Γ_1_ values, the one-factor model showed excellent model fit (CRMR = 0.03, Γ_1_ = 1 for both analyses).

As expected, the results from the principal axis factoring (*N* = 43) were less robust. Although acquisition, morphine/cue-induced reinstatement and stress/cue-induced reinstatement showed positive factor loading values (all |loadings| ≥ 0.27), the loading for elasticity of demand was outside of theoretical bounds with a |loading| = 1.03 (Table 1). Since factor loadings in the standardized 1-factor model can be interpreted as correlations, values outside of the −1 to 1 interval are indicative of a Heywood case. As noted earlier, this mathematically illogical result can occur when common factor extraction methods are applied to small sample data sets. The fact that the principal axis method produced a Heywood case in our data provides further justification for our choice to use robust methods for factor extraction. Therefore, it was not surprising that the one-factor principal axis solution did not fit the data well as indicated by CRMR = 0.07.

## Discussion

Our data demonstrated that a single latent addiction factor fits four distinct MSA measures. This indicates that acquisition, elasticity of demand, morphine/cue-induced reinstatement, and stress/cue-induced reinstatement all in some way measure a common construct, akin to a general factor of addiction vulnerability. These findings support the implicit assumption in the preclinical literature that these different SA measures are related to abuse liability. This one-factor model is also consistent with the clinical literature that often posits a single latent factor to underlie multiple measures of addiction (19–21).

In terms of individual factor loadings, results from both regularized FAs implicated elasticity of demand as the variable most reliably strongly associated with the addiction factor, with a stable, high factor loading across both analyses. Previous studies have demonstrated the value of behavioral economics in studying individual differences in vulnerability to addiction to opioids and other drugs in both humans and animals (14, 16, 41–43). For example, elasticity of demand predicts a variety of other measures of cocaine and opioid SA in rats (41, 42). The high factor loading for α in the current study complements these findings and further demonstrates the utility of this demand function for studying drug addiction.

In contrast, stress/cue-induced reinstatement did not load onto the addiction factor. Stress-induced reinstatement differed from the other three measures in that it (1) involved stress, which can induce relapse via partially distinct biological mechanisms (44), and (2) was tested in the absence of morphine. To evaluate whether either of these features could account for our findings, we tested an additional model (see Supplemental Materials), in which we added cue-induced reinstatement, and replaced elasticity of demand (α) with intensity of demand (Qo), an alternative behavioral economic measure that reflects the maximum level of consumption at zero price. Neither cue-induced reinstatement or Qo involve acute stress, while Qo is derived from data collected in the presence of morphine. Neither of these measures showed high factor loading, suggesting that neither the presence of stress, nor the absence of morphine, can alone account for the poor loading of stress-induced reinstatement onto the addiction factor. Further research using more complex models is needed to elaborate the factor-analytic structure of MSA. This could include evaluation of whether stress/cue-induced reinstatement, cue-induced reinstatement, and Qo load onto a single, additional latent factor or are each associated with different sources of variance.

Utilizing different regularized FA methods with robust correlations in direct comparison with a traditional principal axis factoring method, we have demonstrated the feasibility of these statistical tools in analyzing sample sizes that are realistic targets for preclinical studies where traditional FA methods might fail. These methods help address some major statistical challenges in small sample size factor analyses such as Heywood cases, which was observed using the traditional factor extraction method (45, 46). Moreover, the regularization methods used in the current study have been shown to provide good recovery of underlying factor structures in simulation data, increasing confidence in the interpretation of our results (29, 30).

Though statistical methods such as regularization enable complex multivariate analyses of small sample sizes, there are inherent limitations of such analyses, such as sampling bias, that could not be fully addressed in this study. Future studies could include a larger preclinical sample for analyses where cross-validation is warranted, such as regularized factor analytic methods using least absolute shrinkage and selection operator (LASSO) penalization (28, 47). Additionally, with a larger preclinical sample, a higher count of observed variables could be included in the model, allowing for examination of more complex multi-factor models.

A further limitation of this study is that some rats had prior morphine and/or naloxone experience, and all rats underwent ICSS surgery and training. However, no significant difference was found on any SA measure between rats with morphine and/or naloxone experience compared to saline controls. Furthermore, despite their history of ICSS testing, rats from the current study showed similar acquisition and demand compared to rats from a previous study that did not have a history of ICSS testing (14, 16). The fixed order of assessment of the MSA outcomes also represents a potential limitation. While some measures (e.g., acquisition) inevitably precede others in a SA model, when possible future studies should counterbalance the phases (e.g., stress-induced and morphine-induced reinstatement) to control for any potential order effects.

Notwithstanding these limitations, the current study represents a first step in using robust FA to understand the factor structure of opioid SA. As such, our study identifies a single factor that contributes to four common opioid SA measures, revealing the common and unique information each of the measures could contribute to preclinical addiction literature. Elasticity of demand most reliably represents the common “addiction” factor. Therefore, future studies examining individual differences in opioid SA may be rendered most informative by selectively examining this variable. More generally, exploring relationships beyond prevailing bivariate correlations in preclinical behavioral studies may further our understanding of addiction vulnerability and its neurobiological basis and lead to better prevention and treatment.

## Data Availability Statement

The original contributions presented in the study are included in the article/Supplementary Material, further inquiries can be directed to the corresponding author.

## Ethics Statement

The animal study was reviewed and approved by Institutional Animal Care and Use Committee (IACUC), Hennepin Health Research Institute.

## Author Contributions

YS contributed to experimental design, conducted behavioral studies on which these findings are based, conducted statistical analyses, and contributed to writing and revision of the manuscript. NW conducted statistical analyses and contributed to revision of the manuscript. JG and AH contributed to experimental design and writing and revision of the manuscript. All authors contributed to the article and approved the submitted version.

## Funding

Supported by NIH/NIDA grant R21 DA037728 (JG and AH), NIDA U01 DA051993 (JG and AH), the Hennepin Healthcare Research Institute (formerly Minneapolis Medical Research Foundation) Translational Addiction Research Program (AH), Hennepin Healthcare Research Institute Career Development Award (AH), and NIDA training grant T32 DA007097 (YS).

## Conflict of Interest

The authors declare that the research was conducted in the absence of any commercial or financial relationships that could be construed as a potential conflict of interest.

## Publisher's Note

All claims expressed in this article are solely those of the authors and do not necessarily represent those of their affiliated organizations, or those of the publisher, the editors and the reviewers. Any product that may be evaluated in this article, or claim that may be made by its manufacturer, is not guaranteed or endorsed by the publisher.
