# Supplementary material for: Individual Differences in Different Measures of Opioid Self-Administration in Rats Are Accounted for by a Single Latent Variable
Source: Front Psychiatry. 2021 Sep 7;12:712163. doi: 10.3389/fpsyt.2021.712163 (PMC8453143; doi:10.3389/fpsyt.2021.712163)
Supplement: Supplementary file 1 [file Data_Sheet_1.pdf]

Supplementary Materials

An alternative model was tested to help understand the findings from our originally hypothesized model. This five-variable, single-factor model retained acquisition, morphine/cue-induced reinstatement and stress/cue-induced reinstatement, added cue-induced reinstatement, and replaced elasticity of demand (α) with an alternate behavioral economic metric, intensity of demand (Q_0). A one-factor model was fitted to the data and analyzed with the same methods described in Methods.

For the two regularized FA analyses, 3 multivariate outliers ($\alpha = 0.1$) were identified from the chi-squared test using Mahalanobis distance. Subsequently, these 3 multivariate outliers were excluded from the robust correlation matrix computation using MCD ($N = 40$). Using the robust correlation matrix with LS estimation, the first regularized FA with LS estimation revealed that acquisition (loading = 0.65), and morphine/cue-induced reinstatement (loading = 0.59) showed high factor loadings on a single common factor. Cue-induced reinstatement showed moderate factor loading (loading = 0.44) whereas intensity of demand (loading = 0.17) and stress/cue-induced reinstatement (loading = 0.33) showed low factor loadings on this dimension. The second FA using ML estimation showed similar results, with acquisition (loading = 0.66) and morphine/cue-induced reinstatement (loading = 0.59) showing high factor loadings, cue-induced reinstatement showing moderate factor loading (loading = 0.45) and stress/cue-induced reinstatement (loading = 0.33) and intensity of demand (loading = 0.17) showing low factor loadings on the latent factor. Overall, the one-factor model showed

poor model fit in this alternative one-factor model (gamma = 0.8, CRMR = 0.17). Poor model fit was also observed using principal axis extraction (CRMR = 0.15).
